# Role of Artificial Intelligence in the Diagnosis of Gastroesophageal Reflux Disease

**DOI:** 10.7759/cureus.62206

**Published:** 2024-06-11

**Authors:** Sravani Kommuru, Faith Adekunle, Santiago Niño, Shamsul Arefin, Sai Prudhvi Thalvayapati, Dona Kuriakose, Yasmin Ahmadi, Suprada Vinyak, Zahra Nazir

**Affiliations:** 1 Medical School, Dr. Pinnamaneni Siddhartha Institute of Medical Sciences & Research Foundation, Vijayawada, IND; 2 Medical School, American University of the Carribbean, Cupecoy, SXM; 3 Surgery, Colegio Mayor de Nuestra Señora del Rosario, Bogota, COL; 4 Internal Medicine, Nottingham University Hospitals NHS Trust, Nottingham, GBR; 5 Medical School, Government Kilpauk Medical College, Chennai, IND; 6 Internal Medicine, Petre Shotadze Tbilisi Medical Academy, Tbilisi, GEO; 7 Medical School, Royal College of Surgeons in Ireland - Medical University of Bahrain, Busaiteen, BHR; 8 Internal Medicine, Wellmont Health System/Norton Community Hospital, Norton, USA; 9 Internal Medicine, Combined Military Hospital, Quetta, PAK

**Keywords:** git endoscopy, ai and machine learning, infectious esophagitis, gastroesophageal reflux disease (gerd), artificial intelligence in medicine

## Abstract

Gastroesophageal reflux disease (GERD) is a disorder that usually presents with heartburn. GERD is diagnosed clinically, but most patients are misdiagnosed due to atypical presentations. The increased use of artificial intelligence (AI) in healthcare has provided multiple ways of diagnosing and treating patients accurately. In this review, multiple studies in which AI models were used to diagnose GERD are discussed. According to the studies, using AI models helped to diagnose GERD in patients accurately. AI, although considered one of the most potent emerging aspects of medicine with its accuracy in patient diagnosis, presents limitations of its own, which explains why healthcare providers may hesitate to use AI in patient care. The challenges and limitations should be addressed before AI is fully incorporated into the healthcare system.

## Introduction and background

Gastroesophageal reflux disease (GERD) stands out as one of the most widespread gastrointestinal conditions worldwide and one of the most diagnosed digestive pathologies in the United States, with a prevalence of more than 20% [1]. According to the 2006 Montreal consensus, GERD is a series of symptoms and complications secondary to regurgitating gastric contents into the esophagus [2]. The clinical presentation of GERD is manifested by typical symptoms such as heartburn, regurgitation, and esophageal chest pain. However, different atypical presentations have been described, including chest pain, dental erosions, chronic cough, laryngitis, or asthma [3].

GERD can be classified into three different phenotypes based on endoscopic and histopathological appearance, including non-erosive reflux disease (NERD), erosive esophagitis (EE), and Barrett's esophagus (BE) [4]. While it is both treatable and preventable, most patients with GERD often do not seek advice from their primary care physician and usually rely on over-the-counter medications. Hence, patients tend to present at later stages of the disease, when complications such as Barrett's esophagus have already set in, warranting superior investigations and treatments, increasing the healthcare burden. GERD could be misdiagnosed as myocardial infarction, which again increases unwanted hospital visits and diagnostic procedures. The scope of artificial intelligence (AI) within GERD lies in developing effective strategies for screening and early diagnosis and its potential complications. 

AI uses computer algorithms that imitate essential human functions like learning and problem-solving. Although it has limitations, AI is more efficient in performing healthcare tasks as compared to the traditional system [5]. There have been studies where AI has been used to depict the anatomical locations in endoscopic images to diagnose GERD accurately [6]. AI has also been used in clinical questionnaires regarding GERD, which are used to diagnose GERD promptly and adequately without invasive procedures. With increasing research being conducted on the use of AI in diagnosing GERD, it also presents its limitations; AI requires a vast amount of data from previous patients to be fed into the system in order to produce more reliable results, it lacks real abstract human-like thinking, and most of the systems available are research-based and may suffer from selection bias [6]. 

With the recent increase in research on the use of AI in diagnosing various GI diseases, healthcare workers would need to further educate themselves on the method of diagnosis, criteria of diagnosis, and advantages and disadvantages of AI use. We reviewed the available studies regarding the use of AI in diagnosing GERD. 

## Review

Technical aspects of AI

**Figure 1:**
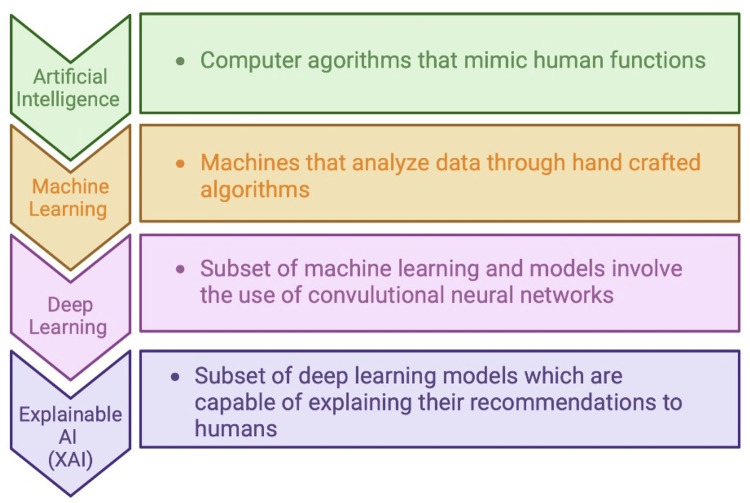
Artificial intelligence (AI) and its subsets Image Credit: Authors; created using biorender.com Reference: [10,11]

AI is a computer algorithm designed to imitate human cognitive processes. Machine learning (ML) and Deep Learning (DL) are the latest advancements in AI. In ML, researchers use their knowledge to identify relevant features in an existing database, which they then use to train a model [5]. The newer and most potential derivative of ML is DL, which autonomously extracts distinctive attributes of input data using artificial neural networks (ANN) [5]. Figure 1 explains AI and its different subsets [7-9]. 

The components of a DL model include pre-processors, feature extractors, classifiers, and explainable AI (XAI). Few studies may include attention modules to fine-tune the feature extractors. Feature extractors are used to convert raw data into a form that the classifier can process. This feature extraction process not only preserves the original data but also enhances the outcome of the AI model [10,11]. There are three types of feature extractors: (a) supervised models that use labeled data to obtain all the general features of an image, e.g., the edges, angular points, texture,s and marginal points of the images, histograms of oriented gradients (HOG), local binary pattern (LBP), scale-invariant feature transform (SIFT), (b) supervised models to obtain the general features of the image using the prior knowledge, and (c) unsupervised feature extraction algorithm that use unlabeled data includes convolutional layers of the convolutional neural network (CNN).

As the information flows through each layer, DL models extract the maximum number of image features from the given input, refining the inferences of the previous layer [12,13]. Classifiers separate the extracted features obtained from the feature extractor into multiple groups including support vector machine (SVM), neural network, gradient boosting classification tree (GB), random forest, K-nearest neighbors (KNN), histogram-based gradient boosting classification tree (HistGB), extreme gradient boosting classification tree (XGB), linear SVM, SVM with radial basis function kernel (RBF), decision tree, Gaussian Naive Bayes, logistic regression, and multilayer perceptron (MLP) [14]. One of the significant concerns of DL models is that the several neuronal layers involved in these models could have explained how the data was interpreted before decision-making. This was considered a black box for DL models [8,9].

**Figure 2:**
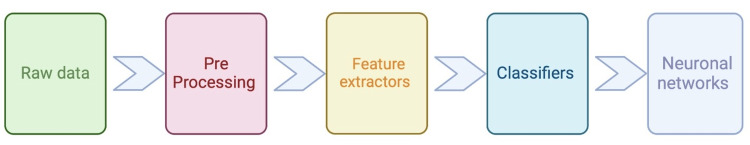
Outline of a basic DL model Image Credit: Authors; Created using biorender.com Reference: [9]

Through continuous innovations, a new subset of DL called interpretable DL or XAI came into existence. These models could explain the rationale behind data interpretation and decision-making [7,10]. Biran and Cotton, in their survey on XAI, mentioned three different types of models: (a) interpretable models, (b) prediction interpretation and justification models, and (c) visualization models [10]. All these models are designed to overcome the bias in the CNNs of the DL models. Figure 2 shows the outline of a basic DL model [9].

While AI was initially viewed as a threat to many healthcare jobs, including physicians, it is certainly not [15,16]. Clinical medicine remains an art that depends on the decisions made by licensed practitioners to ensure transparency and maximum patient safety [15]. In today's existing scenario, AI is only being used as a middle ground to find out things that are not easily identifiable by humans. However, the significance of these findings must still be determined by licensed practitioners [17-20].

Role of AI in diagnosing GERD

One of the initial studies conducted on using AI in the detection of GERD was conducted by Pace et al., who developed the Questionario Italiano Diagnosticos (QUID) questionnaire to distinguish erosive esophagitis from non-erosive GERD [21]. The data was explored further using ANN, a neuronal network implemented to diagnose the type of GERD. The results obtained were statically significant (p<0.001) for GERD patients as compared to the controls, but it failed to distinguish erosive from non-erosive esophagitis [21]. In a study conducted by the Mayo Clinic, all patients enrolled in the study had to fill out a questionnaire suggested by the Mayo Clinic, the Gastro-Esophageal Reflux Questionnaire (GERQ) [22]. These patients underwent an EGD and a 24-hour esophageal pH meter. GERD was objectively confirmed in 103, and it was customary in 56 patients. The AI system automatically selected the 45 most relevant variables from the GERQ (collectively referred to as QUID), allowing the CAD tool to predict GERD in all 103 patients with an accuracy of 100% [22]. 

Endoscopy

GERD is typically diagnosed based on a clinician's assessment of symptoms and treated empirically. According to the current guidelines by the American College of Gastroenterology, endoscopy is the first line of investigation in patients with symptoms of GERD after the initial eight-week trial of proton pump inhibitors (PPIs) [23]. The scope of AI was extended into endoscopy in the form of two popular techniques: computer-assisted detection (CADe) and computer-assisted diagnosis (CADx). With CADe, the AI model acts as a "second pair of eyes" for the investigator, and the CADx system is used to classify a lesion based on several morphological parameters and generate probability scores for malignancy or non-malignancy [23,24]. Although these techniques have been widely used in the past by colonoscopists to increase adenoma detection, similar techniques can be applied to upper GI endoscopy to detect GERD and its other potential complications like Barrett's esophagus and esophageal adenocarcinoma. A pioneering AI model for diagnosing GERD was the hierarchical heterogeneous descriptor fusion (HHDF)-SVM developed by Huang et al. [25]. This method utilized conventional endoscopic images for diagnosis. Their method could automatically diagnose GERD and achieve better accuracy than state-of-the-art methods [24,25]. The initial AI models used in diagnosing GERD, i.e., the QUID questionnaire and HHDF-SVM, were limited to differentiating patients with GERD from healthy patients but failed to classify GERD. 

AI Models Used to Classify GERD

One of the first AI models to classify GERD according to the Los Angeles classification was proposed by Wang et al., a DL model with data augmentation GERD-VGGNet that used convolutional neural networks to classify GERD [26]. They selected 2000 cases, of which only 496 images passed after quality checks. Images from 464 patients were used for model training and validation, and the remaining 32 images were used to evaluate and compare the accuracy of both trainees and the proposed model. Among the feature extractors that were compared in this study to extract the image features, the VGG 16 model showed 100% training accuracy and 89.3 validation accuracy. So, upon combining VGG 16 with classifiers (GERD-VGGNet model, RBF-SVM, Decision Tree, Random Forest, and Adaboost), the GERD-VGGNet and VGG 16 combination model outperformed others. The development set's model demonstrated an overall accuracy of 99.2% (grade A-B), 100% (grade C-D), and 100% (standard group) when employing narrow-band image (NBI) endoscopy. Meanwhile, on the test set, the suggested model achieved an accuracy of 87.9% [26]. 

Another similar model that aimed to classify GERD using narrow-band imaging (NBI) endoscopic images was proposed by Yen et al. at Chung Shan Medical University Hospital in 2022 [14]. The model was developed using 671 NBI endoscopy images in the development set and 32 images in the test set. The development set consisted of 244 grade A-B images, 229 grade C-D images, and 198 standard endoscopic images, which were classified based on the Los Angeles classification of GERD. The model combined both DL and ML aspects of AI. GerdNet (feature extractor)-RF(classifier) was developed to process the NBI images. The model performed much more efficiently than the GERD-VGGNet model. The overall accuracy increased from 98.9±0.5% to 99.0±0.1%, and the test accuracy also improved from 78.8±8.5% to 92.5±2.1%.

A study was conducted at the Hanoi Medical University Hospital, Vietnam, to develop a method using data augmentation techniques to classify GERD from endoscopic images. ResNet-50 was used for feature extraction and classification. Two data augmentation techniques, Affine Transformation and Generative Adversarial Network (i.e., StyleGAN2-ADA) on different color models (RGB, HSV), were used to improve the classification of GERD due to which there was a significant improvement from 83.2% to 91.7% in accuracy when classifying GERD using StyleGAN-ADA on RGB channels compared to the original data on the dataset [27]. 

Another study in Xiangya Hospital of Central South University, Hunan, China, added to the use of AI to diagnose GERD. The main aim of the study was to provide a framework for the diagnosis of Barrett's esophagus and reflux esophagitis in endoscopic images using DL techniques. Experimental results showed that this model achieved a classification recognition accuracy of 93.0%, with macro-precision, macro-recall, and macro-F scores of 93.5%, 92.9%, and 93.2%, respectively [28]. 

Proceeding with more models and comparison studies, we reviewed a study by Liao et al. aimed to compare the classification performance of the regions of interest (ROI)-based CNN models with the LBP-based SVM classifier, the HOG-based SVM classifier, and the SIFT-based SVM classifier [12]. All these were AI-based models for endoscopy. They used a dataset containing 1394 clinical NF-NBI images from 50 subjects, of which 554 images were from 21 patients (NERD+) and 840 were from 29 healthy controls. They observed that in both the subject-dependent and subject-independent experiments, the ROI-based CNN models achieved a higher mean of 10-fold test accuracy compared to the SVM classifiers, with a 20.5% improvement in the former and a 14.0% improvement in the latter. This underscores the superior classification performance of the ROI-based CNN models over the SVM classifier [12]. 

More recent additions to the use of AI came to light when a DL model was developed that classified endoscopic images based on the Los Angeles grade [29]. This study compared the performance of XAI models. A total of 2081 endoscopic images were obtained. The compared models are Grad-CAM, information bottlenecks for attribution (IBA), and an intrinsic (Scouter) method. Among the feature extractors and classifiers, dense 121 outperformed the others. So, dense 121 was experimented for comparing the XAI models. In this visualization experiment, the Scouter method and the Grad-CAM had the best w-Precision (weighted precision) and best incremental area under the curve (iAUC), respectively. In terms of the efficiency of the DL model, the classification accuracy in Grade A and Grade C was significantly higher than that of endoscopists. This model showed an accuracy of 86.7% when compared with endoscopists [29].

One of the most recent studies was by Ge et al., which compared the performance of classification by the DL models with that of endoscopists using Hill's grading system [30]. Their study included 3256 samples. The test data set included 333 samples, and the rest of the samples were imparted into training and validation data sets. ResNet-50 and Xception outperformed the other models among the feature extractors and classifiers. Post-feature extraction, the data was subjected to early dynamic attention (ED-A), which primarily worked on the color intensity of the RGB image, and SE attention, which worked on the spatial orientation of the image. Grad-CAM algorithm was used as the XAI technique for visual interpretation. The experiment revealed that attention modules help the model to localize ROI, enhancing classifying abilities. ResNet-50 combined with attention modules performed the best, as is evident from the area under the curve (AUC) reaching 0.989 and an accuracy of 93.39% [30].

A significant long-term complication of chronic untreated GERD is a premalignant condition called Barrett's esophagus. Early detection of neoplasia in these patients can result in improved treatment outcomes and a reduction in healthcare costs. In 2019, de Groof et al. developed the CADe model to differentiate neoplastic and non-dysplastic lesions in patients with Barrett's esophagus [31]. Using this model, they evaluated five independent endoscopy data sets. They found that the CADe system achieved superior results with higher accuracy than 53 nonexpert endoscopists. The model classified images as containing neoplasms or nondysplastic Barrett's esophagus with 89% accuracy, 90% sensitivity, and 88% specificity [31]. In 2020, another DL model was proposed by Hashimoto et al. [32] for early visual detection of esophageal neoplasia in patients. The evaluation encompassed 455 test images, with the detection of early neoplasia yielding a sensitivity of 96.4%, specificity of 94.2%, and accuracy of 95.4%.

One of the main challenges of upper digestive endoscopy is unseen areas which are usually missed by trainee endoscopists referred to as blind spots. Wu et al. proposed a DL model named ENDOANGEL with an aim to decrease the number of blind spots. This AI model is currently approved by the FDA [33]. Although initially used to detect early gastric cancer as a secondary output, this model could be extended to detecting esophageal cancer. In their study, 1050 patients were randomized into the ENDOANGEL group (n=498) and a control group (n=504), and it was found that the ENDOANGEL group had fewer blind spots (mean 5.38, SD 4.32 vs. 9.82, SD 4.98; P < 0.001). 

On a side note, de novo GERD can occur after sleeve gastrectomy, a bariatric procedure performed in morbidly obese individuals. A meta-analysis published in 2021 that included 46 studies totaling 10,718 patients stated that the increase of postoperative GERD after sleeve gastrectomy was 19% and de novo reflux was 23% [34]. An ML model was proposed in 2022 to detect the probability of postoperative GERD in sleeve gastrectomy patients by utilizing the SPSS software (IBM Corp., Armonk, New York, United States [35]. The study revealed that the model had excellent accuracy with an AUC of 0.93, yet a moderate sensitivity of 79.2% and specificity of 86.1%. 

A lesser-known extraesophageal manifestation of gastroesophageal reflux disease, laryngopharyngeal reflux (LPR) occurs when stomach contents flow backward into the laryngopharynx. This is associated with hoarseness, vocal fatigue, excessive throat clearing, globus pharyngeus, chronic cough, postnasal drip, and dysphagia [36]. Laryngoscopic findings may include erythema, edema, ventricular obliteration, postcricoid hyperplasia, and pseudosulcus change [37]. A unique study, which deviated from the routine endoscopic investigations and impedance studies to diagnose GERD, was conducted at Mayo Clinic, Rochester, which aimed to use voice recordings to distinguish Barrett's esophagus from GERD using ML algorithms [38]. They used a sample of 245 patients (98 vocally normal, 34 GERD patients, and 35 with Barrett's esophagus). Patients categorized as GERD+ exhibited erosive esophagitis (Los Angeles grade B-D), peptic stricture, or acid exposure time exceeding 6%. Barrett's esophagus was delineated as columnar mucosa invasion surpassing 1 cm, accompanied by confirmed specialized intestinal metaplasia. Patients lacking these criteria were classified as GERD-. Voice recording consisted of a six-sentence standard script read over 25-45 seconds from patients undergoing esophagogastroduodenoscopy (EGD) and ambulatory pH monitoring studies due to clinical indications. Random forest models were used as feature extractors and classifiers. They used an F1 (0-100) score to interpret the results of their study and found that the proposed AI had an excellent ability to distinguish Barrett's esophagus and GERD from normal voice, with scores ranging from 60 to 70 [38].

Impedance studies

The gold standard for determining whether GERD is pathological is pH impedance testing; however, manual analysis takes a long time. AI can streamline the interpretation of pH-impedance studies [38]. Normal individuals, patients with previous GERD, and patients with esophageal functional disorders have higher baseline impedance values than those with erosive esophagitis and NERD, and low mean nocturnal baseline impedance can independently predict the efficacy of anti-reflux medical or surgical treatment [39]. Acid exposure time (AET) is the most critical parameter obtained from impedance monitoring in diagnosing GERD. According to the Lyon Conesus, AET < 4% is physiological for GERD, and AET > 6% is pathological. However, AET between 4% and 6% is determined to be inconclusive and warrants further evaluation. The other metrics that should be evaluated in such cases include several reflux episodes, mean nocturnal baseline impedance (MNBI), and reflux-induced peristaltic wave (PSPW). AI advances have been made to study the number of reflux episodes, MNBI, and PSPW index, but no significant AI application was developed to measure the AET [40]. This might be one of the most important missing links in diagnosing GERD and a potential future research advancement. We reviewed the existing literature and the models proposed to enhance the detection and interpretation of baseline impedance values. 

A study was undertaken to develop an AI model across three tertiary medical centers in Taiwan [36]. The aim was to assess the performance of a cascade multivariate long short-term memory fully convolutional network (MLSTM-FCN) model, which outperformed other models and showed an accuracy of 0.917, sensitivity of 0.818, and specificity of 1.00 when compared with experts in the field [36].

A new research project, carried out jointly by the University of Wisconsin-Madison and Peking University Third Hospital, employed end-to-end DL techniques for diagnosing LPR [41]. According to the pH monitoring results, 37 subjects were diagnosed with LPR, while 38 subjects showed symptoms suggestive of LPR but tested negative. The control cohort comprised 77 individuals who were deemed healthy. Proximal acid episodes, proximal acid exposure time, impedance detected proximal acid exposure, and the reflux score index (RSI) were used to categorize data. ResNet-18, ResNet50, and ResNet101 were feature extractors and classifiers. The study discovered that ResNet50 integrated with the CBAM module achieved the highest classification accuracy, reaching 73.4%. Meanwhile, ResNet18 equipped with the CBAM module demonstrated the highest AUC value at 73.9%, outperforming other models [41].

A study conducted in Taiwan utilized 7939 impedance events from 106 patients who underwent pH-impedance studies to establish an AI model [42]. The data for this study was obtained from patients aged 20-65 years with typical GERD symptoms (heartburn and acid regurgitation) for at least three months and negative endoscopy, who underwent 24-hour impedance-pH monitoring without PPI therapy for at least seven days. Time intervals of 30 seconds before the event start time and 90 seconds after the event start time were considered. The raw waveform signals were then transformed into RGB-colored images for a total time of 120 seconds. The raw data was converted into grayscale images for input into the deep residual learning model for image recognition through attention. The feature extractor cum classifier (ResNet18) was used to identify reflux episodes. It demonstrated that the accuracy of the AI model in recognizing reflux episodes was up to 87% [42].

 In conclusion, the ML system is 68.7% sensitive to identifying reflux episodes and 80.8% specific [43]. 

Limitations of AI

AI, while a powerful tool in medicine, is not without its limitations. One of the most significant challenges is the economic and regulatory burden it can place on organizations. The approval of safety, efficacy, and transparency regulations is a prerequisite for the utilization of AI technology in clinical settings [44]. However, these systems require constant updating and upkeep, including the integration of large amounts of data, software updates, and hardware repairs. Such tasks necessitate human resources and funding support, which can pose significant economic and regulatory challenges [45-48]. The high cost of developing, validating, and integrating AI into healthcare systems can be a deterrent, and stringent regulatory standards, while necessary for patient safety, can also slow down the process.

**Figure 3:**
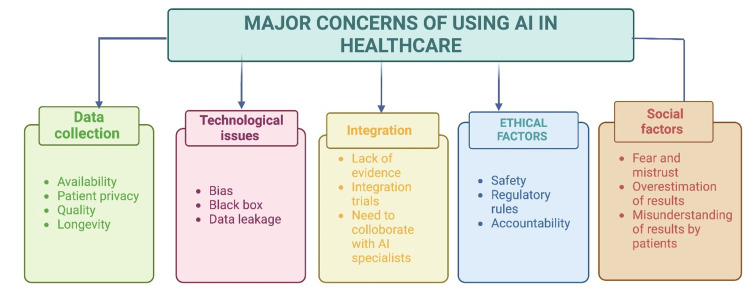
Concerns of AI in healthcare Image Credit: Authors; Created using biorender.com Source reference: [45] AI: artificial intelligence

Additionally, the language used in AI tools can be a limitation. Tools developed in one language may not immediately apply in different linguistic or cultural contexts, necessitating further adaptation and resources to ensure broad usability [47]. Another challenge in AI-assisted medicine is creating a communication channel between the model and physicians. This involves translating digital information into medical language, aiding diagnosis and treatment [24]. Figures 3 briefly summarize the disadvantages of integrating AI into clinical practice [45]. At the same time, AI can reduce backlogs and mundane tasks for healthcare workers and cut costs apart from several other pathbreaking innovations [49] and there needs to be large-scale research to make it more accessible to the end users.

## Conclusions

Integrating AI into the diagnosis process of GERD offers enormous potential to transform clinical practices and patient outcomes positively. AI technologies have found their place in image analysis, interpretation, and classification of GERD, which can give valuable insights to help in early detection, accurate diagnosis, and tailored treatment. However, it is crucial to understand the limitations and challenges that may arise. Addressing these challenges through continued research, development, and training is essential for harnessing the full capabilities of AI to improve patient outcomes in GERD management. 
